# The complete chloroplast genome of a Korean endemic ornamental plant *Hosta yingeri* S. B. Jones (Asparagaceae)

**DOI:** 10.1080/23802359.2017.1398608

**Published:** 2017-11-09

**Authors:** Chae Eun Lim, Soonok Kim, Hyun Oh Lee, Se-A Ryu, Byoung-Yoon Lee

**Affiliations:** a National Institute of Biological Resources, Incheon, Korea;; b Department of Phylogenomics, Phyzen Genomic Institute, Seongnam, Korea

**Keywords:** *Hosta yingeri*, chloroplast genome, Asparagaceae, phylogenetic analysis

## Abstract

*Hosta yingeri* is a perennial herbal ornamental plant belonging to the Asparagaceae family and an endemic species distributed in islands of Korea. In this study, complete chloroplast genome sequence of *H. yingeri* was characterized through *de novo* assembly with next generation sequencing data. The chloroplast genome is 156,756 bp in length and contains four rRNA genes, 30 tRNA genes, and 77 protein-coding genes. Phylogenetic analysis demonstrated a close relationship of *H. yingeri* with other species belonging to the subfamily Agavoideae in Asparagaceae.

*Hosta yingeri* S. B. Jones belongs to the *Hosta* subgenus *Bryocles*, and is an endemic species of Korea (Maekawa 1940; Schmid 1991; National Institute of Biological Resources 2013). The species is very rare in distribution, and only a few populations are found in off-shore islands of southwestern region in Korea such as Daeheuksan-do, Hong-do, Soheuksan-do (Chung 2007; Chung and Kim 1991). *Hosta yingeri* differs from other species in the genus by having relatively thick, adaxially shiny dark green leaves, delicate raceme of flowers spread evenly around the ventral axis of the inflorescence and exceptional length of the second set of stamens (Jones 1989). Since its horticultural potential, approximately 20 cultivars originated from *H*. *yingeri* are being cultivated and traded in the United States and Europe (http://www.hostalibrary.org). On the other hand, genus *Hosta* is well known for its difficulty in the classification (Bailey 1930; Stearn 1931; Hylander 1954). Although there have been several attempts to determine the boundaries between taxa and to understand interspecific relationships (Zonneveld and Iren 2001; Sauve et al. 2005), the criteria for delimiting the species remain unclear. In addition, origin of the many *Hosta* cultivars on the market is uncertain.

In this study, we determined the complete chloroplast (cp) genome of *H*. *yingeri* to contribute to the classification and development of DNA markers for authentication of *Hosta* species or cultivars. The specimen was collected from Heuksan-do (34°40′35.7″ N, 125°25′25.5″ E), Shinan-gun, Jeollanamdo, South Korea, and deposited in National Institute of Biological Resources with the accession number NIBR-VP0000632797. Sequencing was conducted using the Illumina MiSeq platform (Illumina Inc., San Diego, CA) and high quality paired-end reads of ca. 1.4 Gb were assembled into a circular DNA (GenBank accession no. MF990205). The chloroplast genome was 156,756 bp in length with 37.8% overall GC content. The genome structure was similar to the reported chloroplast genome of closely-related species *H*. *ventricosa* (McKain et al. 2016), and contained 77 protein-coding genes, 30 tRNA genes, and four rRNA genes. The chloroplast genome was composed of a large single copy (LSC), a small single copy (SSC), and two inverted repeat (IR) regions of 85,116 bp, 18,232 bp, and 26,704 bp, respectively. Total 20 genes (eight tRNAs, four rRNAs, and eight protein-coding genes) were duplicated in inverted repeat regions. On the other hand, the *rps*16 gene in the cpDNA of *H*. *yingeri* was pseudogenized by the absence of exon no. 2, as in the cpDNAs of other Asparagaceae species reported thus far (McKain et al. 2016). In addition, the *infA* and *ycf15* genes were also lost in *H. yingeri*. To understand its phylogenetic status within Asparagaceae, a maximum likelihood (ML) tree was constructed using 72 protein-coding genes of *H*. *yingeri*, 22 species from Asparagales, four species from Liliales, and two outgroup species (Figure 1). The ML tree showed that *H. yingeri* has a closely relationship with *H*. *ventricosa*. In addition, the chloroplast genome data also supported that Genus *Hosta* is belonging to the subfamily Agavoideae in Asparagaceae (APG IV 2016).

**Figure 1.**
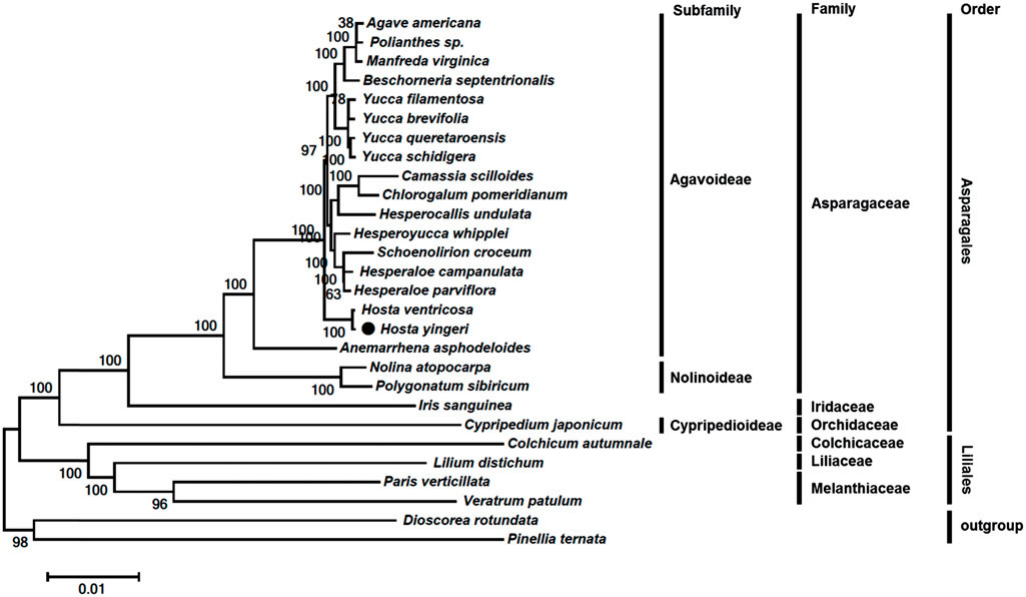
Maximum-likelihood tree based on the chloroplast protein-coding genes of 28 taxa including *Hosta yingeri*. Sequences of 72 chloroplast protein coding genes from order Asparagales and Liliales as well as two outgroup taxa were aligned using MAFFT (http://mafft.cbrc.jp/alignment/server/index.html) and used to generate maximum-likelihood phylogenetic tree by MEGA 6.0 (Tamura et al. 2013). The numbers in the nodes indicated the bootstrap support values (>50%) from 100 replicates. Chloroplast genome sequences used for this tree are *Agave americana*, KX519714; *Anemarrhena asphodeloides*, KX931449; *Beschorneria septentrionalis*, NC_032699; *Camassia scilloides*, NC_032700; *Chlorogalum pomeridianum*, NC_032701; *Colchicum autumnale*, KP125337; *Cypripedium japonicum*, KJ625630; *Hesperaloe campanulata*, NC_032702; *Hesperaloe parviflora*, NC_032703; *Hesperocallis undulata*, NC_032704; *Hesperoyucca whipplei*, NC_032705; *Hosta ventricosa*, KX931460; *Hosta yingeri*, MF990205; *Iris sanguinea*, KT626943; *Lilium distichum*, KT376489; *Manfreda virginica*, NC_032707; *Nolina atopocarpa*, KX931462; *Paris verticillata*, KJ433485; *Polianthes sp.*, KX931464; *Polygonatum sibiricum*, KT695605; *Schoenolirion croceum*, NC_032710; *Veratrum patulum*, KF437397; *Yucca filamentosa*, KX931467; *Yucca brevifolia*, NC_032711; *Yucca queretaroensis*, NC_032713; *Yucca schidigera*, NC_032714; *Dioscorea rotundata*, KJ490011 (Dioscoreales; Dioscoreaceae, as outgroup); *Pinellia ternata*, KR270823 (Alismatales; Araceae, as outgroup).
